# Study Based on Gamification of Tests through *Kahoot!™* and Reward Game Cards as an Innovative Tool in Physiotherapy Students: A Preliminary Study

**DOI:** 10.3390/healthcare11040578

**Published:** 2023-02-15

**Authors:** Irene Cortés-Pérez, Noelia Zagalaz-Anula, María del Carmen López-Ruiz, Ángeles Díaz-Fernández, Esteban Obrero-Gaitán, María Catalina Osuna-Pérez

**Affiliations:** Department of Health Sciences, University of Jaen, Campus Las Lagunillas s/n, 23071 Jaen, Spain

**Keywords:** health teaching, learning, gamification, *Kahoot!*, reward cards, innovation, physiotherapy, education in health sciences, motivation, academic performance

## Abstract

Background: *Kahoot!* is an educational tool allowing teachers to create a series of gamified tests with the aim of reinforcing educational content, thus improving the teaching-learning process. The objective of this project is to evaluate the acquisition of content through gamified tests with *Kahoot!* and reward cards compared to the traditional teaching methodology (contents not reinforced). Methods: This Physiotherapy Teaching Innovation Project (PTIP) was carried out in four subjects of the Degree in Physiotherapy at the University of Jaén (Spain). The teachers responsible for each subject were instructed in the use of *Kahoot!* and reward cards. These teachers randomly selected the contents that were going to be reinforced with *Kahoot!* while the other 50% of the contents would not be reinforced. In the final exam of each subject, the results related to the reinforced contents were compared with those non-reinforced and the degree of satisfaction of the students with the experience was evaluated. Results: A total of 313 students participated in this PTIP. In all subjects, we determined a significant increase in the number of correct answers in an improvement range from 7% (95% CI 3.85 to 9.38) to more than 20% (95% CI 17.61 to 26.86) in favor of the questions that alluded to reinforced content using *Kahoot!* compared to the non-reinforced contents. More than 90% of the participants considered the use of *Kahoot!* useful and motivating. Our findings showed that *Kahoot!* motivated more than 65% of students to study daily. Conclusions: The students obtained better academic results in the questions related to contents reinforced with tests through *Kahoot!* and reward cards compared to those non-reinforced, showing that this methodology can be an effective tool to promote retention and content assimilation.

## 1. Introduction

At present, we live with technological and creative resources that are increasingly complete, attractive, easy to use and highly useful for almost all aspects of life, including education [1]. The new learning strategies suggest that the student-centered approach has more satisfactory results in their learning ability and academic performance compared to more traditional methodologies [2]. In teaching, the introduction of mobile devices and gamification methods is giving rise to new forms of interaction between teachers and students [3]. The term gamification refers to the application of the usual game resources (dynamics, designs, elements, etc.) in non-recreational contexts to encourage participation and favor the teaching–learning process and problem solving through actions that increase the motivation [4]. With the application of gamification in the classroom, interactive teaching and group work are promoted, improving the teaching–learning process [5]. Recent studies postulate in favor of educational technology based on gamification in the educational field, encouraging students to become more involved in their learning [6,7]. Some web and mobile applications and patient simulators in virtual environments allow the incorporation of gamification in the teaching process of health degrees, allowing the incorporation of clinical simulations with a high degree of realism [8,9]. On the other hand, the inclusion of gamification as innovative teaching material requires an evaluation of its effectiveness as a learning tool, since it is sometimes difficult to incorporate this methodology depending on the curriculum of the subject or the characteristics of the students and if the teaching is face-to-face or online [10]. According to some studies, the attention of the students in the master classes decreases to a great extent around the 18th minute, hence the need to create new, more creative and playful content that can increase their interest [11]. Much of the teaching staff seeks to enhance the creativity of their students, encourage participation in classes and motivate them; however, many of the teaching resources used (PowerPoint or Keynote slides, clinical cases, project-based learning, among others) are not so attractive to the teacher. New students, on the contrary, are very familiar with new technologies [12].

Recent studies have suggested the need for a change in the university educational system currently oriented towards a more theoretical aspect than the teaching of professional care [13]. In this sense, teachers encounter difficulties due to the increase in the number of university students and the lack of resources [14]. From this aspect arises the need to involve students in a more active and dynamic learning that does not focus on lectures. This new learning will allow interaction with the teacher in the form of brainstorming, debates or open-ended reasoning questions, thus favoring learning based on participation that increases motivation [15,16]. Motivation is a very important element in the teaching–learning process, directly linked with it, stimulating the student in a positive way and encouraging them to participate in the activities proposed in the classroom [17]. When a good gamified structure is presented in a class, students experience a playful feeling that spontaneously reinforces their learning, which could increase their academic performance [18]. Gamification is understood as a form of motivation, although difficult to maintain, on many occasions motivated by internal and external agents to the subject itself [19,20], hence the need to innovate and create didactic content that attracts students [21].

An example of teaching gamification is the use of the mobile or web application *Kahoot!™* created by Professor Alf Inge Wang, which is a partially free platform that allows students to be involved in the teaching–learning process through questionnaires or predesigned surveys (testing procedures) [22]. To use *Kahoot!*, teachers must create a user account on the web platform of this tool. From there, questionnaires or tests can be developed on the online platform with up to two (true or false questions) or four response alternatives covering different contents. The program allows you to insert videos, photos and music into these tests to encourage students or simply to bring upbeat and motivating energy to the test. Students do not need to create a user account on *Kahoot!*, they simply access this platform with their smartphone or computer with a code provided by the teacher, being able to carry out, at that moment, the preselected activity (test). In addition, *Kahoot!* carried out for students can be revisited sometimes with the aim to review the contents again. Students mark the answer option that they consider correct at the same time motivated by having a predetermined limited time and knowing in advance that success and speed of response are rewarded [23]. At the end of the test, the platform stands as a podium, with the three winning students being able to contribute to positive learning [24]. In addition, responses from all participating students are automatically collected in the *Kahoot!* teacher’s account, and can be reviewed later in a Microsoft Excel document provided by the web platform. This allows to measure the understanding of the contents by the students and to obtain optimal feedback on the correct development of the subject. The results obtained in *Kahoot!* can be used for a wide variety of assessments and projects, including formative assessments, research projects, and study presentations [25,26]. On the other hand, another widely used gamification method is game cards with or without rewards, which allow the activity to be associated with classic board games, once again providing a playful and motivating environment for learning [27].

Finally, the level of student satisfaction is another factor to take into account in the teaching–learning process; therefore, it must be carefully studied in gamified environments. In this sense, student satisfaction can be defined as “a short-term attitude that results from an evaluation of the educational experience, services and facilities” [28]. Several studies have agreed that students who had positive experiences in school and were satisfied with their teaching–learning experience reported higher levels of mental and physical health, higher academic performance, and greater overall satisfaction with their lives [29]. The combination of *Kahoot!* together with the game cards, which are associated with a kind of recognition, prize or reward for the students who score the most correct answers in the activity, is an interesting and promising association in the educational experience that this study will attempt to analyze in university students studying physiotherapy. Therefore, the objective of this study is to evaluate the acquisition or assimilation of the contents taught through tests using *Kahoot!* along with reward-based game cards as a gamification tool to reinforce academic content in students studying physiotherapy. Secondarily, we also intended to analyze the level of satisfaction and motivation of the students in relation to the use of *Kahoot!* and reward-based playing cards.

## 2. Materials and Methods

### 2.1. Study Design

This study used an experimental design [30]. This study was assessed positively by the Vicerrectorado de Coordinación y Calidad de las Enseñanzas (Coordination Office and Quality of Teachings) at the University of Jaén with the following identification number: PIMED35_201921. Before that project began, participants were informed of all details and they were asked for consent. The confidentiality and anonymity of the data of each participant was guaranteed throughout the entire study with the Organic Law 7/2021 of 26 May.

### 2.2. Study Context

The framework for this research is comprised by different subjects of the Degree in Physiotherapy at the University of Jaén (Jaén, Spain). In Spain, the Degree in Physiotherapy is obtained over the course of 4 years comprising 240 ECTS (that correspond to approximately 6000 h of training). More specifically, there were four subjects participating in this research: Physiotherapy Fundamentals (first-year course), Kinesitherapy (second-year course), Special Massage Therapy (third-year course) and Abdomino-pelvic Physiotherapy (fourth-year course). The choice of subjects was made for convenience between those taught on that date in the Physiotherapy Degree, according to the teaching Organization, and those in which the teaching staff agreed to participate in the study. The information of each subject is summarized in Table 1.

### 2.3. Participants

All the students enrolled in each subject participated in this Physiotherapy Teaching Innovation Project (PTIP). The inclusion criteria to participate in this research were as follows: (1) students enrolled in the Degree of Physiotherapy from University of Jaén; (2) currently taking one of the four subjects participating in this PTIP; and (3) participating voluntarily and being able to report on the various dimensions of the PTIP in the subject that they are enrolled in. The researchers who participated in this PTIP were the professors responsible for each subject, experts with more than 4 years of teaching experience in the Degree of Physiotherapy at the University of Jaén. In total, 319 persons participated in the study. On the one hand, 313 students were the sample evaluated (Physiotherapy Fundamentals, *n* = 76; Kinesitherapy, *n* = 85; Special Massage Therapy, *n* = 81; and Abdominopelvic Physiotherapy, *n* = 71). On the other hand, six teachers participated in coordinating and developing the project and analyzing statistical data.

### 2.4. Description of the Physiotherapy Teaching Innovation Project Using Kahoot! and Reward Game Cards

This PTIP was carried out between 2019 and 2022, in the 2019/2020, 2020/2021 and 2021/2022 academic courses. The development of this project coincided with the restrictions imposed by the COVID-19 pandemic. However, during home confinement, the lessons were conducted online, and *Kahoot!* (code and testing questions) could be displayed in the computer or smartphone screen of the students, permitting them to perceive the questions. The objectives of this project are shown in Table 2.

**Figure 1:**
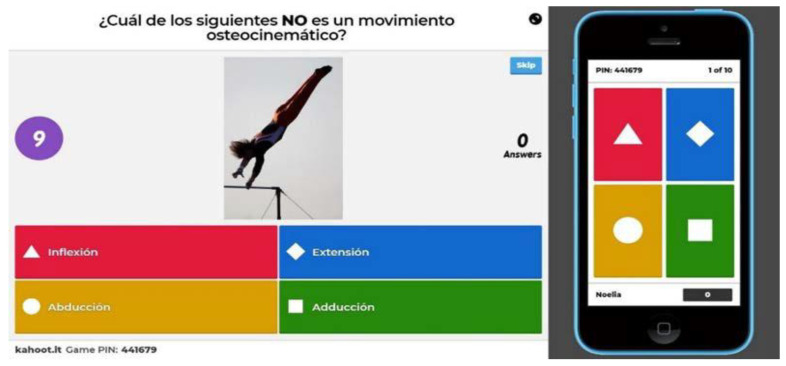
*Kahoot!* Test projected in class (left) and appearance of the student’s smartphone (right) during the period given to select the correct answers. Image obtained from a self-made questionnaire on the *Kahoot!* platform.

To carry out this PTIP, the participating teachers had to learn to use the *Kahoot!* tool to create gamified tests and download data in Excel from the platform. Quizzes created by *Kahoot!* are presented in game mode, in which the student’s mobile device or laptop becomes a remote control with four buttons, each one of a different color and with a different geometric shape (red—triangle, blue—rhombus, yellow—circle and green—square). Each question in the questionnaire presents four response options, and each option appears with a symbol and color corresponding to each of the four buttons in order to select the desired option. Therefore, from the electronic device, it is possible to select an answer to the question that the teacher has launched from his computer, projector or electronic whiteboard (Figure 1). Each teacher committed to creating their own *Kahoot!* questionnaires with content from their subjects. Each teacher prepared for the 50% of the theoretical contents of their subject *Kahoot!* tests which had to have at least 8 questions with 4 answer alternatives. In this way, half of the subject content was reinforced with gamification and the other half followed the traditional teaching model (without reinforcement).

**Figure 2:**
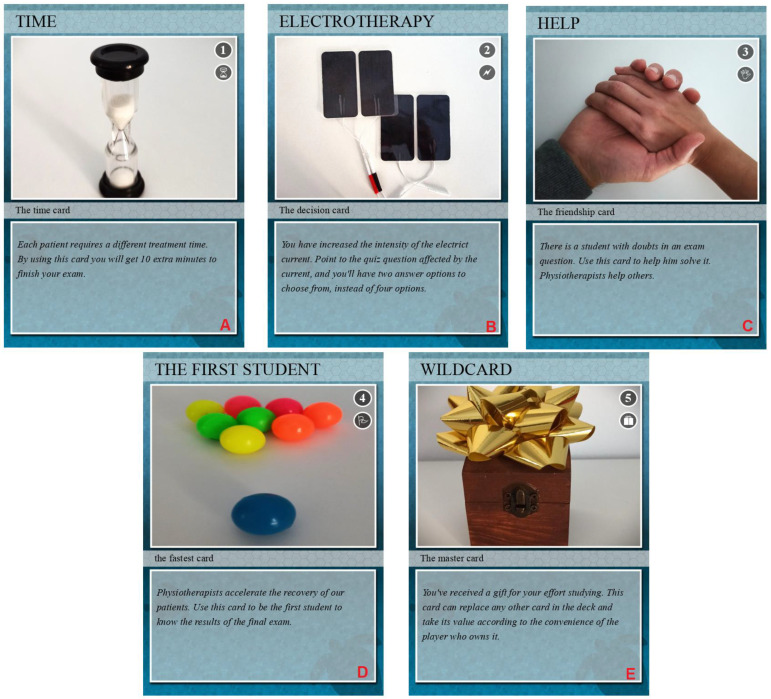
Reward-based playing cards using in this PTIP: (**A**) the time card; (**B**) the decision card; (**C**) the friendship card; (**D**) the fastest card; (**E**) the master card.

Next, the teachers participating in the project designed reward-based playing cards to reward the winners of each *Kahoot!* session. The objective was to increase the motivation of the students to study the subject, attend class and participate to win their cards. The game card won (which is a recognition of the study and learning in the subject) must help the winning student to improve their score for the final grade of the subject. The cards were designed to resemble board game cards using the Big Huge Labs applications available at https://bighugelabs.com/deck.php, accessed on 23 June 2020. Figure 2 shows the reward-based playing cards used in this PTIP.

Once the teachers learned to use *Kahoot!* and create the game cards, the teacher responsible for each subject selected the theoretical content to be taught. The theoretical contents of each subject were taught for 30 h over a period of four months. Of all the theoretical contents of the subject, 50% were randomly selected to be reinforced with *Kahoot!* and game cards. Each randomly selected piece of theoretical content had to be reinforced with a test *Kahoot!* of 8 questions with 4 answer alternatives. In this way, half of the subject was reinforced using *Kahoot!* and game cards and the other half followed the traditional teaching model (without reinforcement). The *Kahoot!* tests which were carried out in teaching of the 50% of the theoretical contents of each subject were carried out at the end of the theoretical explanation of each block with the aim of reinforcing the learning of the recently explained contents. In addition, after answering each question in *Kahoot!*, the teacher resolved any doubts about that question, as well as explained the reasons why one question was true and the other three were false, providing instant feedback to the students. The student who obtained the first position in each *Kahoot!* received a reward-based game card. The objective of this methodology was to compare if the contents reinforced with *Kahoot!* were better received than those taught with traditional teaching methods. The compare the results, the teacher downloaded the results of the reinforced content quizzes off the *Kahoot!* platform, which were compared with the results of those based on non-reinforced content obtained in the final written exam. The final exam for each subject was a multiple choice quiz that consisted of 40 questions, 20 referred to contents reinforced with gamification and another 20 to non-reinforced contents.

Finally, the level of satisfaction with the experience was also evaluated. For this, an ad hoc self-developed questionnaire was used to assess that evaluated the perception of the students about the *Kahoot!* platform and game cards as a teaching–learning innovation tool, the innovation of the project, the motivation, the usability and the general satisfaction with this PTIP.

### 2.5. Data Analysis

Data handling and analysis were conducted using the statistical package for social sciences (SPSS) version 21 (SPSS Inc., Chicago, IL, USA). The results were presented using descriptive statistics. The correct question scores were expressed as mean, standard deviation (SD) and in percentages (%). Differences in the distribution of the responses between reinforced contents and non-reinforced contents were analyzed with Student *t*-test for each final exam. Statistical analysis was conducted considering *p* < 0.05 statistically significant and with a level of confidence of 95% (95% CI).

## 3. Results

### 3.1. Effect of Kahoot! on Study Contents

**Figure 3:**
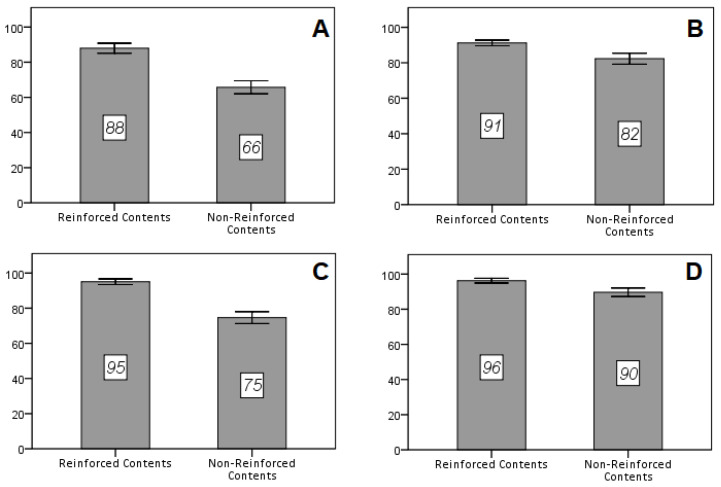
Percentages of correct answers in the final exam of (**A**) Physiotherapy Fundamentals; (**B**) Kinesitherapy; (**C**) Special Massage Therapy and (**D**) Abdominopelvic Physiotherapy subjects. Reinforced contents vs. non-reinforced contents.

In all courses, a significant difference was detected in the number of correct answers in the final test exam of each subject (Table 3, Figure 3). An improvement in the scores and in the number of correct answers to the questions that alluded to reinforced contents was determined. Specifically, the greatest difference was observed in the subjects of the first- and third-year course. More than 20% of correct answers in average terms (95% CI 17.61 to 26.86; *p* < 0.001 for the first-year course and 95% CI 16.69 to 24.17; *p* < 0.001 for the third-year course) was obtained in the reinforced contents compared with non-reinforced contents. In the second- and fourth-year courses, the improvement in the percentage of correct answers was statistically significant but with less impact, almost 9% and 7%, respectively (95% CI 5.43 to 12.33; *p* < 0.001 for the second-year course and 95% CI 3.85 to 9.38; *p* < 0.001 for the fourth-year course). These results allow us to consider gamification (based on the *Kahoot!* application and reward cards, in this case) as a powerful didactic tool that effectively contributes to the learning process and content assimilation.

### 3.2. Students’ Satisfation with Kahoot! Experince as an Educative Tool

At the end of the project, the students completed the satisfaction survey on one of the last days of class. A total of 232 participants completed the questionnaire. The global results and the results in each subject are presented in Table 2.

In all the questions, the answers of “strongly agree” or “agree” resulted in the highest percentages among the participants of the different courses. More than 90% of the total participants considered that games with *Kahoot!* helped them to review concepts of the subject and clarify doubts; the students also think that gamification might be useful in other subjects. Approximately 90% of participants considered it motivating that *Kahoot!* quizzes were linked to reward cards and considered *Kahoot!* an innovative tool for teaching and learning. *Kahoot!* activities have motivated more than 65% of students to study every week (not only for the final exam). Almost all the participants were able to participate in the *Kahoot!* activities from home when the classes were online. Finally, in general, the students showed a very high or moderate degree of satisfaction with the experience. The results of the satisfaction questionnaires are shown in Table 4.

## 4. Discussion

This study showed that a post-lecture activity of testing with *Kahoot!* and reward cards affected retention of lecture contents over a 15-week retention interval as measured by a final exam. In addition, we determined that taking several previous short answer tests through both gamification techniques produced significantly better retention of the material than taking the traditional teaching methodology. In addition, the students positively assessed, in general, the inclusion of these new teaching tools in the development of the classes. The degree of participation was very high in all the subjects, mainly due to the high percentage of student attendance in class, and none of them refused to participate. Comparable studies that applied gamification techniques obtained similar participation rates [31,32,33]. The total number of students in the study was high (*n* = 313) since a large part of the students of the entire Physiotherapy Degree participated. Therefore, it is a higher sample than that of most related studies [32,34,35]. Regarding the study duration, it was approximately 15 weeks (the period in which the complete teaching of each one of the subjects was carried out), more than enough time to regularly apply the teaching tools at the end of classes and in line with other related studies [36,37,38].

The primary finding was that taking multiple choice tests through gamification (*Kahoot!* combined with reward cards) produced superior retention of lecture contents after almost four months relative to the traditional teaching method.

There is considerable scientific evidence in cognitive psychology that testing can improve the retention of explained material in the classroom through a post-class testing procedure that can be easily implemented in the classroom [39,40]. In addition to boosting retention, frequent testing can help to lower students’ test anxiety and increase the regularity of studying [41]. Moreover, many studies have determined that taking a test produces greater material retention than re-studying of the material [42,43]. The testing effect is definitely a robust and reliable phenomenon demonstrating that taking an initial test improves performance on subsequent tests [44]. In line with this and subsequent finding [45], we applied the *Kahoot!* game after the subject was taught, as the repetition at the end of the lesson provides reinforcement and facilitates better knowledge retention related to the content. Finally, we decided to choose multiple-choice tests as the post-lecture reinforcement since the final exam will be conducted in the same manner. 

The results were statistically significant in all the courses analyzed (students of different age and knowledge groups) and in different subjects, which suggests that they may be independent of these factors and that the best academic performance was due to the combined use of the tools of gamification and reward. Both techniques motivated and reinforced the students to assimilate the contents, regardless of what they were. Furthermore, the standard deviation of the correct answers in the group of contents reinforced by gamification was also lower in all courses, indicating that the results were grouped around the mean with little dispersion, showing a more consistent and stable academic performance. Furthermore, the standard deviation of the correct answers in the group of contents reinforced by gamification was also lower in all courses, indicating that the results were grouped around the mean with little dispersion, showing a more consistent and stable academic performance and a reflection of the *Kahoot!* app’s ability to boost accuracy in responses. Our goal was not to promote competitiveness, but to make the learning process exciting. The latest studies in neuroeducation indicate that it is difficult to learn without conscious and sustained attention. In addition, it is known that attention is aroused when teaching is different, gamified and curious [12].

The most significant effect of this positive reinforcement learning through gamification and reward was obtained in the first and third-year courses. The specific characteristics of each subject or teacher can explain this result.

These promising results could be explained by the fact that the innovative techniques motivate students to attend classes and be more engaged with them [46,47,48]. Students are usually excited and motivated to experiment with different technologies while learning, mainly because they have skills in operating mobile technologies and enjoy using mobile apps and games. The learning benefits of the *Kahoot!* web platform are well documented by numerous studies [31,34,36,46,48,49,50,51,52,53]. Furthermore, these tools also motivate students taking online courses, where the lack of attention is frequent [54] and there is a need to focus on and engage with the class [55,56].

The design of the studies about gamification in education is very heterogeneous. In some cases, there are case studies or quasi-experimental studies (without a control group) with a test–retest design [32,40,51,55,57,58], and some of them compare academic results of previous courses with results after gamified teaching [59,60]. In our study, the research team decided on the correct response rate of reinforced content with these gamification tools as the primary variable to encourage all students to participate and benefit from the innovative learning method.

Although the methodology of our study differs from that implemented in many of the studies, we have observed similar results with an improvement in students’ academic results in most of them [31,32,37,40,46,53,59,60,61,62]. Castro et al. (2019) developed a study with a methodology similar to our research [32]. Their results showed that the questions previously answered using *Kahoot!* in the classroom were significantly easier questions for students in the final exam. These findings are consistent with our study that showed significantly better scores in questions on the reinforced contents by *Kahoot!* combined with reward cards than those that were not reinforced. 

In all cases, feedback was provided after responses (the correct answer was given or explained) to improve retention [63]. As results are shown immediately, students can discuss results and solve doubts forthwith [64] in a safe environment [65]. In addition, feedback should also be provided to ensure students learn from the test, especially in the event of poor performance.

During the study, at some point, almost all the students had to attend class and gamification activities online due to the COVID-19 regulations in effect at that time, which compelled students to take rotating shifts in classes to keep a safe distance at all times. The student–teacher interaction was remote and synchronous, and it was possible through the application of Google Meet; the students were able to use gamification tools with no issues reported. However, very few studies analyze this tool in online classes [33,66], and our findings are consistent with them. Predictably, due to reduced face-to-face class contact because of COVID-19, further research in health education and the use of *Kahoot!* will substantially increase [46,67].

Response time in *Kahoot!* quiz games is a relevant element because it is essential for solving problems in the clinical practice of healthcare professionals [32,68]. Therefore, one of the main advantages of using *Kahoot!* in class is that it provides a ranked score based on the number of correct answers and response time, promoting a certain degree of competitiveness among students. The implementation of educational games which consider response time and correct answers favors competitiveness, motivates students to actively participate in their learning process [32] and positively affects students’ academic results [24].

Although the vast majority of the studies show favorable results of using *Kahoot!* as a teaching tool compared to traditional methods, some evidence does not support this. The reasons these authors provided to justify these results were diverse. For example, distractions due to visual effects [69], less direct interaction with the teacher to ask specific questions [70], little time to answer questions [32], students not used to or who did not like mobile games presenting more anxiety with the quiz competition [71] or little intervention time and new content that needed more time to assimilate [72] were the unfavorable aspects. Therefore, to further enhance the possible positive effect of gamification with *Kahoot!*, the research team proposed introducing another motivation element into the study: reward cards. The students who managed to appear on the leaderboard of the *Kahoot!* were gifted with a reward card, representing direct benefits for the student in the subject’s final exam (e.g., extra time or elimination of incorrect answers in any question chosen by the student themself). Although some studies explore the effects of digital rewards [73,74], we have not detected evidence in university studies of the type of reward tool we have used in ours.

Both gaming activities motivated more than 65% of students to study every week, and not only on the days before the final exam, changing the students’ study behavior. Surprisingly, this aspect has not received much attention in previous studies [75], although it may be an essential element of academic success.

In general, students showed a high level of satisfaction with using these game-based tools in the classroom; these results are consistent with related research [23,32,34,35,36,46,76]. The highest-rated options in the survey were “*The games with Kahoot! helped me to review concepts of the subject*” and “*The games with Kahoot! have helped me to clarify doubts*”, with more than 90% of the students choosing these options [47,51,57,76]. In addition, approximately 90% of the students agreed with the following: “*I think that games with Kahoot! might be useful in other subjects*” [51]. In other words, students asserted that *Kahoot!* increased teacher–student interaction and was a valuable tool in the learning process. Our results suggest that students positively evaluated the use of educational games and considered games helpful learning strategies that could capture their attention and engage them in the learning process [32,77].

As a strength of our study, it should be noted that much of the scientific literature on the impact of higher education with tools such as *Kahoot!* has been widely studied in numerous subjects of various university degrees and other related health disciplines such as nursing and medicine [1,23,60,70,72,76,78,79]. Unfortunately, however, there is a lack of literature regarding the use of *Kahoot!* in physiotherapy education.

In addition, as a newer and more innovative aspect, the integration in the present study of reinforcement techniques (testing) through gamification (*Kahoot!*) and the extra motivation of reward cards with the hypothesis of improving content assimilation should be noted. So far, we have not detected any other study that brought together these valuable teaching tools, neither in university studies related to Physiotherapy nor other studies from other disciplines.

This study also has two limitations to highlight. The first is due to the methodological design; our primary variable of results was the correct answer rate in the final exam of the contents reinforced after class with the gamification and reward tools. In most related studies, the primary variable is the student’s academic performance (most of them quasi-experimental studies without a control group) [46] usually with a pre-test and post-test evaluation [31,36,57,69,71]. The research team chose this design to provide all students access to the gamification web tool and the reward system without denying opportunities to any student. In addition, the correct answer rate in the reinforced content (either traditionally or gamified) can also be considered a student’s academic result (it is an objectively measured knowledge of the students). The second limitation is that the research team created the general satisfaction scale, designed “ad hoc” for the study. However, many articles related to this topic have also used a similar methodology with surveys developed for this purpose without any additional validation process [31,33,46,55,56,58].

As has been shown in this study, including motivating elements such *Kahoot!* and reward cards can promote the assimilation of concepts, enhance attention in class, and may even improve final academic performance. However, one consideration to keep in mind for future research is whether similar positive results would be obtained only with the implementation of testing with *Kahoot!* but without reward cards. The motivation to obtain subsequent academic benefits in the final content exam could be essential, and without it, it may be that only the use of gamification as reinforcement would not have had such a notable effect. In the same way, other aspects of interest to analyze could be (a) to include the variable “*time of study*” or “*time of content review*” to directly and quantitatively relate the motivation that gamification produces with changes in the student’s study behavior, and (b) to evaluate the effectiveness of gamification tools in cooperative teamwork [76], both in writing the tests and in solving them. However, gamification studies in other educational disciplines have determined improvements in student motivation at an extensive level, enhancing their academic performance [21].

Future research should also focus on learning frameworks with control group studies (even crossover studies) to evaluate these gamification tools as potentially valuable strategies in physiotherapy studies. On the other hand, it would also be interesting to carry out an analysis from the qualitative point of view to determine the individual perceptions of the students.

We believe the present findings have direct implications for educational practice. Our experiment combined ecologically valid presentation materials (lectures) and realistic retention intervals (4 months). Furthermore, the benefits that were determined in this study suggest that the combination of gamification and reward tools can also be helpful for other disciplines, both health and non-health sciences. We encourage these educators to incorporate testing through gamification into their daily classroom routine.

## 5. Conclusions

The students of all the courses of the Physiotherapy Degree obtained better academic results in the questions about contents reinforced with testing through *Kahoot!* and reward cards compared to those that were not reinforced. Test-enhanced learning through these gamification techniques may be an effective tool for health educators to use in promoting the retention of clinical knowledge. Most students who participated in the study valued this new way of learning, especially in reviewing the concepts acquired and clarifying doubts. With a more student-centered approach that motivates them playfully and competitively, this teaching technique represents a powerful and innovative tool that can be used in any subject in Physiotherapy. Furthermore, this new form of teaching interaction can be used in a comfortable and accessible way to improve the learning process and the assimilation of content in university teaching of Health Sciences, in face-to-face or online teaching.

## Figures and Tables

**Table 1:** Subjects of the Degree in Physiotherapy from University of Jaén (Spain) participating in this research.

Subjects’ Name	Course Year	ECTS and Duration (h)	Description
Physiotherapy Fundamentals	First	6 ECTS: 3 ECTS for theory (30 h) and 3 ECTS for practice lessons (30 h)	Compulsory and specific subject that focuses on identifying the concept, evolution and foundations of physiotherapy in its scientific and professional aspect. Students are instructed to understand the general theory of functioning, disability and health and its international classification, as well as the intervention models in physiotherapy and its care practice, as well as know and apply the theoretical bases and the development of physiotherapeutic methods and procedures.
Kinesitherapy	Second	6 ECTS: 3 ECTS for theory (30 h) and 3 ECTS for practice lessons (30 h)	Compulsory and specific subject which addresses the knowledge of the different joints and muscles of the human body, the learning and application of manual and instrumental methods and procedures for passive, active and mechanical mobilization, for therapeutic purposes.
Special Massage Therapy	Third	6 ECTS: 3 ECTS for theory (30 h) and 3 ECTS for practice lessons (30 h)	Compulsory and specific subject that focuses on learning special manual techniques, within the broad scope of Massage Therapy, for specific therapeutic purposes.
Abdominopelvic Physiotherapy	Fourth	6 ECTS: 3 ECTS for theory (30 h) and 3 ECTS for practice lessons (30 h)	Compulsory and specific subject that aims to develop the skills of students in the physiotherapeutic approach to pathology of the abdominal–pelvic–perineal sphere.

Abbreviations: ECTS, European Credit Transfer and Accumulation System.

**Table 2:** Objectives of the Teaching Innovation Project using *Kahoot!*.

Academic Course	Objectives
2019/2020	-To instruct and train the teacher involved in the project to use *Kahoot!*;
	-To teach the teachers involved in the project to design and use reward game cards applied to teaching;
	-To carry out the first pilot tests with *Kahoot!* and reward game cards with students and teachers.
2020/2021 2021/2022	-To implement teaching with gamification in the subjects using *Kahoot!* and reward game cards;
	-Objectively assess the degree of assimilation of the content by analyzing the data from the *Kahoot!* Platform;
	-To check if the contents reinforced using *Kahoot!* are the best evaluated in the global qualification of the subject;
	-To evaluate the degree of satisfaction of the students with the *Kahoot!* and reward game cards experience.

**Table 3:** Effectiveness of gamification in reinforcing content. Correct answers in the final exam of the subject: reinforced contents versus non-reinforced contents.

Academic Grade (Participants, *n*)	Subject	Final Exam Total Questions (Reinforced Contents/Non-Reinforced Contents)	Reinforced Content Correct Answers Mean ± SD (Data in %)	Non-Reinforced Content Correct Answers Mean ± SD (Data in %)	Mean Difference (95% CI) [Data in %]	*p*
First Grade (*n* = 76)	Physiotherapy Fundamentals	40 (20/20)	17.59 ± 2.47 (87.96 ± 12.36)	13.14 ± 3.24 (65.72 ± 16.2)	4.45 (3.52–5.37) [22.24 (17.61–26.86)]	<0.001
Second Grade (*n* = 85)	Kinesitherapy	40 (20/20)	18.24 ± 1.48 (91.18 ± 7.46)	16.46 ± 2.86) (82.29 ± 14.30)	1.76 (1.08–2.47) [8.88 (5.43–12.33)]	<0.001
Third Grade (*n* = 81)	Special massage therapy	40 (20/20)	19.01 ± 1.48 (95.06 ± 7.43)	14.93 ± 3.06 (74.63 ± 15.34)	4.08 (3.34–4.83) [20.43 (16.69–24.17)]	<0.001
Fourth Grade (*n* = 71)	Abdomino-pelvic physiotherapy	40 (20/20)	19.25 ± 1.14 (96.27 ± 5.71)	17.93 ± 2.05 (89.65 ± 10.29)	1.32 (0.77–1.88) [6.62 (3.85–9.38)]	<0.001

Abbreviations: SD, Standard deviation; 95% CI, 95% Confidence Interval; %, Percentage; *p*, *p*-value.

**Table 4:** Results of the satisfaction questionnaire in each course of the Physiotherapy Degree.

Questions		First-Year Course *n* = 48 (%)	Second-Year Course *n* = 64 (%)	Third-Year Course *n* = 80 (%)	Fourth-Year Course *n* = 40 (%)	Total *n* = 232 (%)
The games with *Kahoot!* have helped me to review concepts of the subject	Strongly Agree	39.6	40	71.3	84.6	58
	Agree	56.3	56.9	28.8	15.4	40.3
	Disagree	4.2	3.1	0	0	1.7
	Strongly Disagree	0	0	0	0	0
The games with *Kahoot!* have helped me to clarify doubts.	Strongly Agree	29.2	39.1	43.8	80	45.7
	Agree	58.3	42.2	52.5	20	45.3
	Disagree	12.5	15.6	3.8	0	8.2
	Strongly Disagree	0	3.1	0	0	0.9
I think that games with *Kahoot!* might be useful in other subjects.	Strongly Agree	66.7	45.3	77.5	82.5	67.2
	Agree	29.2	32.8	22.5	12.5	25
	Disagree	2.1	12.5	0	5	4.7
	Strongly Disagree	2.1	9.4	0	0	3
*Kahoot!* activities have motivated me to study every week (not only for the final exam).	Strongly Agree	31.3	25	31.3	50	32.8
	Agree	37.5	28.1	36.3	35	34.1
	Disagree	31.3	28.1	28.8	10	25.9
	Strongly Disagree	0	18.8	3.8	5	7.3
I found it motivating that *Kahoot!* quizzes were linked to reward cards.	Strongly Agree	50	53.1	62.5	80	60.3
	Agree	29.2	32.8	31.3	7.5	27.2
	Disagree	20.8	7.8	6.3	10	10.3
	Strongly Disagree	0	6.3	0	2.5	2.2
I found the use of *Kahoot!* innovative for teaching and learning.	Strongly Agree	39.6	40.6	60	70	52.2
	Agree	56.3	32.8	33.8	27.5	37.1
	Disagree	4.2	21.9	6.3	2.5	9.5
	Strongly Disagree	0	4.7	0	0	1.3
I have been able to participate from home in the *Kahoot!* activities.	Always	77.2	65.6	75	70	72
	Often	22.9	29.7	22.5	30	25.9
	Rarely	0	3.1	2.5	0	1.7
	Never	0	1.6	0	0	0.4
My overall satisfaction with the *Kahoot!* activities and reward cards.	Very Satisfied	47.9	46	60	55	52.8
	Moderately Satisfied	37.5	33.3	33.8	37.5	35.1
	Slightly Satisfied	14.6	15.9	6.3	7.5	10.8
	Dissatisfied	0	4.8	0	0	1.3

## Data Availability

Not applicable.

## References

[1] van Gaalen A.E.J., Brouwer J., Schönrock-Adema J., Bouwkamp-Timmer T., Jaarsma A.D.C., Georgiadis J.R. (2021). Gamification of health professions education: A systematic review. Adv. Health Sci. Educ..

[2] Walker A., Leary H. (2009). A Problem Based Learning Meta Analysis: Differences across Problem Types, Implementation Types, Disciplines, and Assessment Levels. Interdiscip. J. Probl. Learn..

[3] Gould D.J., Terrell M.A., Fleming J. (2008). A Usability Study of Users’ Perceptions Toward a Multimedia Computer-Assisted Learning Tool for Neuroanatomy. Anat. Sci. Educ..

[4] Deterding S., Sicart M., Dk M., Nacke L., O’hara K., Dixon D. Gamification: Using Game Design Elements in Non-Gaming Contexts General Terms. Proceedings of the CHI’11 Extended Abstracts on Human Factors in Computing Systems.

[5] Prensky M. (2006). “Don’t Bother Me Mom, I’m Learning!”: How Computer and Video Games Are Preparing Your Kids for Twenty-First Century Success and How You Can Help!.

[6] Obrero-Gaitán E., Nieto-Escamez F., Zagalaz-Anula N., Cortés-Pérez I. (2021). An Innovative Approach for Online Neuroanatomy and Neuropathology Teaching Based on 3D Virtual Anatomical Models Using Leap Motion Controller During COVID-19 Pandemic. Front. Psychol..

[7] Nieto-Escamez F.A., Roldán-Tapia M.D. (2021). Gamification as Online Teaching Strategy During COVID-19: A Mini-Review. Front. Psychol..

[8] Akl E.A., Sackett K.M., Erdley W.S., Mustafa R.A., Fiander M., Gabriel C., Schünemann H. (2013). Educational games for health professionals. Cochrane Database Syst. Rev..

[9] Hernández E., Camacho M., Leal-Costa C., Ruzafa-Martínez M., Ramos-Morcillo A.J., Cazorla E., Díaz-Agea J.L. (2021). Does Multidisciplinary Team Simulation-Based Training Improve Obstetric Emergencies Skills?. Healthcare.

[10] Hampton D., Pearce P.F., Moser D.K. (2017). Preferred Methods of Learning for Nursing Students in an On-Line Degree Program. J. Prof. Nurs..

[11] Bunce D.M., Flens E.A., Neiles K.Y. (2010). How long can students pay attention in class? A study of student attention decline using clickers. J. Chem. Educ..

[12] Fragkaki M., Mystakidis S., Dimitropoulos K. (2022). Higher Education Faculty Perceptions and Needs on Neuroeducation in Teaching and Learning. Educ. Sci..

[13] Barcelo J.M. (2016). Medical laboratory science and nursing students’ perception of academic learning environment in a Philippine university using Dundee Ready Educational Environment Measure (DREEM). J. Educ. Eval. Health Prof..

[14] Johanna Cedeño Tapia S., Nerly Villalobos Guiza M., Isidro Rodríguez López J., Andrea Fontal Vargas P. (2021). La educación de enfermería en Latinoamérica y los entornos virtuales de aprendizaje en tiempos de pandemia. Rev. Cuid..

[15] Nishimura A. (2018). Effects of different methods of reflection on nurses’ gaze and judgement in a task using a touch panel. J. Clin. Nurs..

[16] Ali R.A., Alnatour A., Alnuaimi K., Alzoubi F., Almomani M., Othman A. (2018). Effects of interactive teaching on university students’ knowledge and attitude toward reproductive health: A pilot study in Jordan. J. Multidiscip. Healthc..

[17] Gopalan V., Bakar J.A.A., Zulkifli A.N., Alwi A., Mat R.C. (2017). A review of the motivation theories in learning. AIP Conf. Proc..

[18] Bouchrika I., Harrati N., Wanick V., Wills G. (2021). Exploring the impact of gamification on student engagement and involvement with e-learning systems. Interact. Learn. Environ..

[19] Ryan R.M., Deci E.L. (2007). Teoría de la Autodeterminación: Necesidades Psicológicas Básicas en Motivación, Desarrollo y Bienestar.

[20] Ryan R.M., Deci E.L. (2020). Intrinsic and extrinsic motivation from a self-determination theory perspective: Definitions, theory, practices, and future directions. Contemp. Educ. Psychol..

[21] Ferriz-Valero A., Østerlie O., García Martínez S., García-Jaén M. (2020). Gamification in Physical Education: Evaluation of Impact on Motivation and Academic Performance within Higher Education. Int. J. Environ. Res. Public Health.

[22] Pintor Holguín E., Gargantilla Madera P., Herreros Ruiz Valdepeñas B., López del Hierro Casado M. (2014). Kahoot en docencia: Una alternativa practica a los clickers. XI Jorn. Int. Innovación Univ. Educ. Para Transform..

[23] Bryant S.G., Correll J.M., Clarke B.M. (2018). Fun With Pharmacology: Winning Students Over With Kahoot! Game-Based Learning. J. Nurs. Educ..

[24] Corell A., Regueras L.M., Verdú E., Verdú M.J., De Castro J.P. (2018). Effects of competitive learning tools on medical students: A case study. PLoS ONE.

[25] Aguiar-Castillo L., Hernández-López L., De Saá-Pérez P., Pérez-Jiménez R. (2020). Gamification as a motivation strategy for higher education students in tourism face-to-face learning. J. Hosp. Leis. Sport Tour. Educ..

[26] Maciej S., Becker F.G., Cleary M., Team R.M., Holtermann H., The D., Agenda N., Science P., Sk S.K., Hinnebusch R. (2019). Online Gamified Training for Business Innovation: Examining an Embodied Gamified E-learning Module on Creativity. J. Creat. Bus. Innov..

[27] Tran L.K., Lipp M.J. (2022). Making competency-based predoctoral orthodontics fun: Introducing Dealodontics. J. Dent. Educ..

[28] Salinda-Weerasinghe I., Lalitha R., Fernando S. (2017). Students’ Satisfaction in Higher Education Literature Review. Am. J. Educ. Res..

[29] Scott Huebner E., Gilman R., Reschly A.L., Hall R. (2012). Positive Schools. Oxford Handbook of Positive Psychology.

[30] Creswell J.W., Creswell J.D. (2017). Research Design: Qualitative, Quantitative, and Mixed Methods Approaches.

[31] Göksün D.O., Gürsoy G. (2019). Comparing success and engagement in gamified learning experiences via Kahoot and Quizizz. Comput. Educ..

[32] Castro M.-J., López M., Cao M.-J., Fernández-Castro M., García S., Frutos M., Jiménez J.-M. (2019). Impact of educational games on academic outcomes of students in the Degree in Nursing. PLoS ONE.

[33] Martín-Sómer M., Moreira J., Casado C. (2021). Use of Kahoot! to keep students’ motivation during online classes in the lockdown period caused by COVID-19. Educ. Chem. Eng..

[34] Wang A.I., Tahir R. (2020). The effect of using Kahoot! for learning–A literature review. Comput. Educ..

[35] Zhang Q., Yu Z. (2021). A literature review on the influence of Kahoot! On learning outcomes, interaction, and collaboration. Educ. Inf. Technol..

[36] Aljezawi M., Albashtawy M. (2015). Quiz game teaching format versus didactic lectures. Br. J. Nurs..

[37] Tóth Á., Lógó P., Lógó E. (2019). The Effect of the Kahoot Quiz on the Student’s Results in the Exam. Period. Polytech. Soc. Manag. Sci..

[38] Moro C., Phelps C., Stromberga Z. (2020). Utilizing serious games for physiology and anatomy learning and revision. Adv. Physiol. Educ..

[39] Butler A.C., Roediger III H.L. (2007). Testing improves long-term retention in a simulated classroom setting. Eur. J. Cogn. Psychol..

[40] Iwamoto D.H., Hargis J., Taitano E.J., Vuong K. (2017). Analyzing the efficacy of the testing effect using KahootTM on student performance. Turk. Online J. Distance Educ..

[41] Leeming F.C. (2002). The exam-a-day procedure improves performance in psychology classes. Teach. Psychol..

[42] McDaniel M.A. (2007). Applying cognitive psychology to education. Psychon. Bull. Rev..

[43] Roediger H.L., Karpicke J.D. (2006). Test-Enhanced Learning. Psychol. Sci..

[44] Chan J.C.K., McDermott K.B. (2007). The testing effect in recognition memory: A dual process account. J. Exp. Psychol. Learn. Mem. Cogn..

[45] ümit YAPICI İ., Karakoyun F. (2017). Gamification in biology teaching: A sample of Kahoot application. Turk. Online J. Qual. Inq..

[46] Donkin R., Rasmussen R. (2021). Student perception and the effectiveness of Kahoot!: A scoping review in histology, anatomy, and medical education. Anat. Sci. Educ..

[47] Jamil Z., Fatima S.S., Saeed A.A. (2018). Preclinical medical students’ perspective on technology enhanced assessment for learning. JPMA.

[48] Wang A.I. (2015). The wear out effect of a game-based student response system. Comput. Educ..

[49] Lamb R.L., Annetta L., Firestone J., Etopio E. (2018). A meta-analysis with examination of moderators of student cognition, affect, and learning outcomes while using serious educational games, serious games, and simulations. Comput. Hum. Behav..

[50] Zhonggen Y. (2019). A meta-analysis of use of serious games in education over a decade. Int. J. Comput. Games Technol..

[51] Aktekin N.Ç., Çelebi H., Aktekin M. (2018). Let’s Kahoot! Anatomy. Int. J. Morphol..

[52] Bakhuys Roozeboom M., Visschedijk G., Oprins E. (2017). The effectiveness of three serious games measuring generic learning features. Br. J. Educ. Technol..

[53] Gentry S.V., Gauthier A., L’Estrade Ehrstrom B., Wortley D., Lilienthal A., Tudor Car L., Dauwels-Okutsu S., Nikolaou C.K., Zary N., Campbell J. (2019). Serious gaming and gamification education in health professions: Systematic review. J. Med. Internet Res..

[54] Rabayah K.S., Amira N. (2022). Learners’ engagement assessment in e-learning during the COVID-19 pandemic: Nation-wide exploration. Educ. Inf. Technol..

[55] Youhasan P., Raheem S. (2019). Technology enabled formative assessment in medical education: A pilot study through Kahoot. Educ. Med. J..

[56] Lohitharajah J., Youhasan P. (2022). Utilizing gamification effect through Kahoot in remote teaching of immunology: Medical students’ perceptions. J. Adv. Med. Educ. Prof..

[57] Neureiter D., Klieser E., Neumayer B., Winkelmann P., Urbas R., Kiesslich T. (2020). Feasibility of Kahoot! as a real-time assessment tool in (histo-) pathology classroom teaching. Adv. Med. Educ. Pract..

[58] Bicen H., Kocakoyun S. (2018). Perceptions of students for gamification approach: Kahoot as a case study. Int. J. Emerg. Technol. Learn..

[59] Fuster-Guilló A., Pertegal-Felices M.L., Jimeno-Morenilla A., Azorín-López J., Rico-Soliveres M.L., Restrepo-Calle F. (2019). Evaluating impact on motivation and academic performance of a game-based learning experience using Kahoot. Front. Psychol..

[60] Strickland H.P., Kaylor S.K. (2016). Bringing your a-game: Educational gaming for student success. Nurse Educ. Today.

[61] Kinder F.D., Kurz J.M. (2018). Gaming strategies in nursing education. Teach. Learn. Nurs..

[62] Krishnamurthy K., Selvaraj N., Gupta P., Cyriac B., Dhurairaj P., Abdullah A., Krishnapillai A., Lugova H., Haque M., Xie S. (2022). Benefits of gamification in medical education. Clin. Anat..

[63] Larsen D.P., Butler A.C., Roediger H.L. (2008). Test-enhanced learning in medical education. Med. Educ..

[64] De Gagne J.C. (2011). The impact of clickers in nursing education: A review of literature. Nurse Educ. Today.

[65] Toothaker R. (2018). Millennial’s perspective of clicker technology in a nursing classroom: A Mixed methods research study. Nurse Educ. Today.

[66] Nuci K.P., Tahir R., Wang A.I., Imran A.S. (2021). Game-based digital quiz as a tool for improving students’ engagement and learning in online lectures. IEEE Access.

[67] Singhal S., Hough J., Cripps D. (2019). Twelve tips for incorporating gamification into medical education. MedEdPublish.

[68] Da Silva J.B., Andrade M.H., de Oliveira R.R., Sales G.L., Alves F.R.V. (2018). Tecnologias digitais e metodologias ativas na escola: O contributo do Kahoot para gamificar a sala de aula. Rev. Thema.

[69] Rondon S., Sassi F.C., Furquim de Andrade C.R. (2013). Computer game-based and traditional learning method: A comparison regarding students’ knowledge retention. BMC Med. Educ..

[70] Courtier J., Webb E.M., Phelps A.S., Naeger D.M. (2016). Assessing the learning potential of an interactive digital game versus an interactive-style didactic lecture: The continued importance of didactic teaching in medical student education. Pediatr. Radiol..

[71] Aras G.N., Çiftçi B. (2021). Comparison of the effect of reinforcement with question-answer and kahoot method on the success and motivation levels of nursing students: A quasi-experimental review. Nurse Educ. Today.

[72] Schultz K., Klein M., Sucharew H., McDonald J., DeBlasio D., Cooperstein E., Poynter S., Huggins J., Real F.J. (2022). The Impact of a Gamified Curriculum Using Kahoot! on Musculoskeletal Knowledge and Skill Acquisition Among Pediatric Residents. Acad. Pediatr..

[73] Joseph M.A., Natarajan J., Buckingham J., Al Noumani M. (2021). Using digital badges to enhance nursing students’ attendance and motivation. Nurse Educ. Pract..

[74] Garnett T., Button D. (2018). The use of digital badges by undergraduate nursing students: A three-year study. Nurse Educ. Pract..

[75] Lameris A.L., Hoenderop J.G., Bindels R.J., Eijsvogels T.M. (2015). The impact of formative testing on study behaviour and study performance of (bio)medical students: A smartphone application intervention study. BMC Med. Educ..

[76] Felszeghy S., Pasonen-Seppänen S., Koskela A., Nieminen P., Härkönen K., Paldanius K.M.A., Gabbouj S., Ketola K., Hiltunen M., Lundin M. (2019). Using online game-based platforms to improve student performance and engagement in histology teaching. BMC Med. Educ..

[77] Boctor L. (2013). Active-learning strategies: The use of a game to reinforce learning in nursing education. A case study. Nurse Educ. Pract..

[78] Díez-Pascual A.M., García Díaz M.P.G. (2020). Audience Response Software as a Learning Tool in University Courses. Educ. Sci..

[79] Barnes R. (2017). Kahoot! in the Classroom. Nurse Educ..

